# Comparing Meta-Analyses with ChatGPT in the Evaluation of the Effectiveness and Tolerance of Systemic Therapies in Moderate-to-Severe Plaque Psoriasis

**DOI:** 10.3390/jcm12165410

**Published:** 2023-08-20

**Authors:** Xuân-Lan Lam Hoai, Thierry Simonart

**Affiliations:** 1 Department of Dermatology, St Pierre—Brugmann—HUDERF University Hospitals, Université Libre de Bruxelles, 1050 Brussels, Belgium; xlamhoai@gmail.com; 2 Department of Dermatology, Delta Hospital, CHIREC, Université Libre de Bruxelles, 1050 Brussels, Belgium

**Keywords:** psoriasis, artificial intelligence, meta-analysis

## Abstract

Background: Meta-analyses (MAs) and network meta-analyses (NMAs) are high-quality studies for assessing drug efficacy, but they are time-consuming and may be affected by biases. The capacity of artificial intelligence to aggregate huge amounts of information is emerging as particularly interesting for processing the volume of information needed to generate MAs. In this study, we analyzed whether the chatbot ChatGPT is able to summarize information in a useful fashion for providers and patients in a way that matches up with the results of MAs/NMAs. Methods: We included 16 studies (13 NMAs and 3 MAs) that evaluate biologics (n = 6) and both biologic and systemic treatment (n = 10) for moderate-to-severe psoriasis, published between January 2021 and May 2023. Results: The conclusions of the MAs/NMAs were compared to ChatGPT’s answers to queries about the molecules evaluated in the selected MAs/NMAs. The reproducibility between the results of ChatGPT and the MAs/NMAs was random regarding drug safety. Regarding efficacy, ChatGPT reached the same conclusion as 5 out of the 16 studies (four out of four studies when three molecules were compared), gave acceptable answers in 7 out of 16 studies, and was inconclusive in 4 out of 16 studies. Conclusions: ChatGPT can generate conclusions that are similar to MAs when the efficacy of fewer drugs is compared but is still unable to summarize information in a way that matches up to the results of MAs/NMAs when more than three molecules are compared.

## 1. Introduction

Meta-analyses (MAs) and network meta-analyses (NMAs) are crucial for synthesizing the enormous amount of information gathered to answer specific questions and are generally considered some of the best tools for evidence-based practice in medicine as they are based on the findings of multiple studies that were identified in comprehensive, systematic literature searches [1,2,3]. A MA/NMA is an especially valuable form of comparative effectiveness research because it emphasizes the magnitude of intervention effects rather than relying on tests of statistical significance among primary studies [2], but it also has some disadvantages and limitations. MAs/NMAs require complex statistical techniques and a significant amount of time to produce (often as long as 1 year) [4] and are, therefore, rarely updated [5,6]. They may also be affected by quality issues (i.e., loose criteria for choosing the studies to be included, minor deviations from protocol [3], publication towards positive studies, an incomplete set of keywords used, and a wide variation in the strategies used to search in different databases) or even conflicts of interest, resulting in biased and misleading results.

The capacity of artificial intelligence (AI) to aggregate huge amounts of information by automatically extracting the written text of medical papers and converting the text into a more structured set of data is emerging as particularly interesting for processing the volume of information needed to generate MAs [7,8,9]. An AI such as the chatbot ChatGPT (chat generative pre-trained transformer) creates realistic and intelligent-sounding text in response to user prompts. It is a ‘large language model’, a system based on neural networks that learn to perform a task by digesting huge amounts of existing human-generated text [10]. As a language model, ChatGPT processes and generates texts based on the input it has been trained on, which includes a wide variety of sources such as books, articles, websites, and other texts. Some of its features include answering follow-up questions, challenging incorrect premises, rejecting inappropriate queries, and even admitting its mistakes [10]. The software company OpenAI, based in San Francisco, California, released the tool ChatGPT on 30 November 2022. ChatGPT is designed to engage in natural and coherent conversations with users, providing responses that are contextually relevant and often indistinguishable from human-generated text ChatGPT, being a versatile language model, has a wide range of potential applications across various domains, including, among others, content generation, language translation, text summarization, educational support, creative writing, coding assistance, language learning, data analysis, social interaction and health. In this study, we investigated whether ChatGPT could correctly summarize the information from available data on treatment for moderate-to-severe psoriasis and estimate the relative efficacy of biologic and systemic therapies in a reliable manner.

## 2. Materials and Methods

### 2.1. Eligibility Criteria, Information Sources, and Search

We investigated the abilities of ChatGPT to compare the different systemic therapeutic interventions for moderate-to-severe psoriasis. As ChatGPT’s training data have a cut-off date of 2021, and as the AI may, therefore, not have access to the most recent information that has been published after that date [11], we compared the results of ChatGPT’s outputs with MAs/NMAs published in 2021 and 2022. We initially performed a search for MAs/NMAs published on PubMed between January 2021 and May 2023 that investigated and compared the effect of biologic and systemic therapies for moderate-to-severe plaque psoriasis. We focused solely on PubMed abstracts and on open-access data since they are freely available both to the public and for AI. The search and eligibility criteria were limited to human studies published in the English language. For easier comparisons, we excluded MAs/NMAs that focused on the nail, scalp, palmo-plantar, erythrodermic, pustular, and pediatric psoriasis, as well as psoriatic arthritis. We also excluded MAs/NMAs on Janus kinase inhibitors, as well as topical, ultraviolet, and combination treatment. MAs/NMAs based on drugs being compared to placebos were not selected. Two authors independently extracted data and assessed the risk of bias.

### 2.2. Study Selection, Data Collection and Data Items

The conclusions of these MAs/NMAs with the ranking of the investigated drugs with respect to their efficacy and/or tolerance were summarized. We asked ChatGPT, which is directly available on the internet free of charge (at the time of redacting), to assess the efficacy and tolerance of the evaluated drugs in selected MAs/NMAs in order to investigate whether the conclusion of the AI matched those of the MAs/NMAs. The queries were sent to ChatGPT between 15 January 2023 and 30 May 2023. The questions were formulated to ChatGPT in different ways (1) to have an overview of the drugs with the highest efficacy and the best tolerance, (2) to have a ranking of the efficacy of the investigated molecules using the same efficacy and safety outcomes as those of the MAs/NMAs, (3) to verify whether ChatGPT’s answers matched the main conclusions of the MAs/NMAs. To evaluate the coherence and the acceptability of ChatGPT’s answers, the results of the queries were submitted to two clinical experts in the field of psoriasis. The details of the queries and of ChatGPT’s outputs, and the ranking of the evaluated molecules are available in the Supplementary Material. The reproducibility between the conclusions of the MAs/NMAs and ChatGPT’s outputs was analyzed and rated as identical (identical ranking for all investigated drugs), acceptable (similar ranking for at least half of the evaluated molecules), different (different ranking for more than half of the investigated drugs), inconclusive (no drug efficacy or tolerance ranking), or not applicable (not investigated in the MAs/NMAs).

## 3. Results

**Figure 1:**
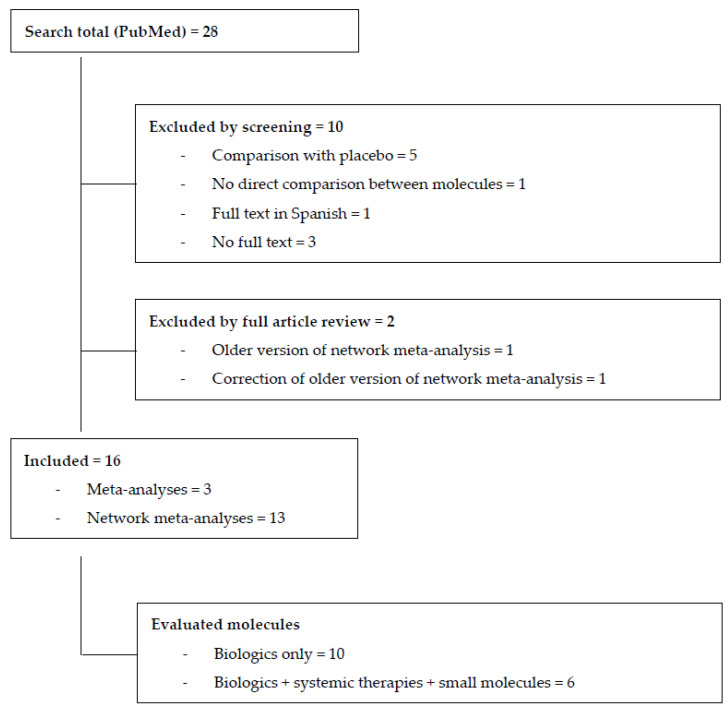
Flowchart of study identification, screening, and inclusion.

We identified 28 MAs/NMAs published between January 2021 and May 2023, investigating and comparing the effect of systemic therapies for moderate-to-severe adult plaque psoriasis [12,13,14,15,16,17,18,19,20,21,22,23,24,25,26,27]. A total of 10 analyses were excluded for the following reasons: insufficient information on the compared therapies in the abstract, the absence of the full text [28,29,30], the full text being in a language other than English [31], being a comparison to a placebo [32,33,34,35,36], or the absence of a direct comparison between therapies [37]. Two older versions or corrections of older MAs were also excluded [38,39] (Figure 1).

The main results of the MAs/NMAs are summarized in Table 1. 

In total, we collected 16 studies, among which 10 focused exclusively on biologics and six evaluated both biologic and systemic treatment (methotrexate, cyclosporin, acitretin, and small molecules). A total of 13 of those 16 selected studies were NMAs, and three were MAs (Figure 1).

Despite significant heterogeneity across all the MAs/NMAs (different evaluated drugs, different numbers of evaluated molecules, different outcome measures, different drug dosages, different ranking methods, different data collection endpoints, and different statistical analyses), there was some consistency in the efficacy and safety rankings of the investigated molecules, with anti-interleukin (IL) 17 and anti-IL23 biologics having the highest short-term and long-term efficacy [13,15,16,17,18,21], and with anti-IL23 biologics generally having the lowest rates of safety events (Table 1) [21].

Depending on the day and time that the queries were sent, ChatGPT’s outputs could vary between vague, general answers and very detailed outputs. We had to reformulate some queries in order to obtain more precise answers, as sometimes general queries did not lead to specific outputs. Depending on the queries, the questions had to be inputted two to five times (mean: 2.5 times) to get an analyzable answer. We chose to select the more detailed answers for easier comparisons with the different MAs/NMAs.

The conclusions of ChatGPT’s outputs were compared to those of the MAs/NMAs and rated in Table 2. Overall, the reproducibility between the conclusions of the MAs and ChatGPT’s results in terms of drug efficacy was rated as identical in 5 out of 16 studies (31%) and acceptable in 7 out of 16 studies (44%) (Table 2). More specifically, the AI generated results that were identical to those of MAs/NMAs in 100% of the cases when three molecules had to be compared (four out of four studies, among which three out of three were MAs). ChatGPT’s outputs were also identical to those of NMAs in 1 out of 13 NMAs (8%). In 4 out of 16 studies (25%), the results were rated inconclusive, as ChatGPT could not generate a specific ranking of the efficacy of the investigated drugs.

The safety of the molecules was compared in two MAs [13,23] and two NMAs [18,21]. Although one meta-analysis indicated that risankizumab was better tolerated than infliximab, ChatGPT’s output was rated as different, as the AI estimated that these two drugs had a similar safety profile. Another meta-analysis showed that risankizumab had a safety profile similar to that of ustekinumab, and the AI’s output was identical. Although two NMAs also showed that anti–IL23 and certain anti-IL17 biologics had lower rates of safety events, the AI’s answers were inconclusive, as ChatGPT could not specifically differentiate the evaluated drugs in terms of safety.

We also noted, in one query, that ChatGPT’s answers contained obvious mistakes, such as presenting infliximab (an anti-TNFα drug) and secukinumab (an anti-IL17 drug) as biologics targeting IL-12 and IL-23, and guselkumab, risankizumab, and tildrakizumab (all anti-IL23 drugs) as anti-IL17A biologics [25]. However, an accurate response was given when the question was rephrased.

More importantly, the whole assessment was completed in less than a few hours over 3 weeks, representing huge time savings compared to the months it usually takes to conduct traditional MAs/NMAs.

## 4. Discussion

The MAs/NMAs on anti-psoriatic drugs combine the results of multiple studies in order to provide a more robust estimate of the relative efficacy of different treatments and are usually generated by experts in the field of psoriasis. However, they require complex statistical techniques and a significant amount of time to produce [4] and may be affected by quality issues. The capacity of an AI to aggregate huge volumes of information by automatically extracting the written text of medical papers and converting the text into a more efficient, structured set of data is emerging as particularly interesting for processing the amount of information needed to generate MAs [7,8,9].

Although some consistency in efficacy rankings was observed for certain drugs across the MAs/NMAs, the rankings for other drugs varied by indirect comparisons. The factors potentially contributing to the heterogeneity of the results of the selected MAs/NMAs include the use of different methodologies for statistical analyses, the variation in drug dosing and treatment duration, the difference in the number and type of evaluated drugs, and the outcome definitions. Many biases may impair the reliability of the conclusions of MAs, and large randomized controlled trials do not always confirm the results of prior MAs [40].

The acceptability and coherence of ChatGPT’s answers could be rated as adequate for most queries by two experts in the field of psoriasis. Although substantial heterogeneity could be evidenced across the MAs/NMAs, the ranking by ChatGPT of the investigated drugs, with respect to their efficacy, was generally comparable to those of the selected studies. When fewer (three) drugs were compared, ChatGPT’s conclusions were identical to those of the MAs/NMAs. It is worth noting that this was valid for MAs published in 2021 or after because ChatGPT’s knowledge cut-off is 2021 [11].

There were, however, some discrepancies between the conclusions of the NMAs and the answers of ChatGPT when several molecules were compared. For instance, although for the majority of the recently published NMAs, ixekizumab and risankizumab [13,15,16,17,18,21] outperformed most biologics in the long-term, secukinumab and guselkumab sometimes ranked as high as ixekizumab and risankizumab in some of ChatGPT’s answers. The choice of reformulating queries or sending them at another timeframe to obtain more detailed answers, although debatable, was deliberate, as the comparisons would have been made impossible if we selected only vague, general outputs.

Despite these mistakes, it appears both exciting and frightening that with an AI-based system, coherent answers to complex questions can be easily obtained within seconds. In addition to its ease and speed of getting results, ChatGPT may be less affected by human bias and potential conflicts of interest than MAs/NMAs in the interpretation of data. The other strengths of ChatGPT are that it might provide more up-to-date information (there are often significant lag times before the publication of MAs) [41], and it might process larger amounts of data from a variety of sources.

ChatGPT relies on open-access data and has no access to the paid content of subscription-based scientific journals. The research community has also recognized a need to incorporate the “grey literature” into MAs to reduce the risks of publication bias (the selective publication of studies based on their results) and reporting bias (selective reporting of study results based on statistical significance) [42]. There is no standard definition of grey literature, but it generally refers to the information obtained from sources other than published, peer-reviewed articles, such as conference proceedings, theses and dissertations, clinical trial registries, adverse events databases, government agency databases (e.g., the US Food and Drug Administration) and documents, unpublished industry data, and online websites, among others. Incorporating grey literature may help to spread the studies with null or negative results that might not otherwise be disseminated [42].

However, in opposition to MAs/NMAs, ChatGPT and other AI are (still) unable to provide a statistical analysis with the corresponding confidence intervals. AI is also (still) unable to address the biases and limitations in individual studies. As ChatGPT’s cut-off knowledge is 2021, its AI may also not have access to the most recent information that has been published or has occurred after that date [11]. Another limitation of ChatGPT is that it lacks transparency concerning the data used to provide answers, as no references are cited in ChatGPT’s answers. There is also the possibility that ChatGPT’s answers may depend on the timeframe they are gathered, which can invalidate the reliability of a dataset. Some of the limitations inherent in MAs (data from heterogeneous sources, publication biases, quality of the source data, etc.) may also be applicable to the data generated by AI. Finally, the large amount of data does not necessarily eliminate sources of systematic error and may even amplify them.

There may also be fears about any reliance on large language models for scientific thinking, as these models are trained on past information, whereas social or scientific progress can often come from thinking or being open to thinking differently from the past. In addition, the widespread use of chatbots such as ChatGPT raises a range of ethical concerns that need to be critically examined (i.e., bias and misinformation, privacy, accountability and liability, manipulation, and malicious use) [43].

To our knowledge, there has so far been no similar attempt to compare the results of MAs/NMAs and the information gathered from ChatGPT. Recently, Anghelescu et al. [44] presented a comparison between a systematic literature review using the PRISMA method—performed by human intelligence—and ChatGPT in order to gather current information on the use of Actovegin in ischemic stroke. They found that the AI-based chatbot could not critically evaluate the quality of evidence, provide a comprehensive analysis of the literature, or provide actual and beyond-question data. On the one hand, they rated the ChatGPT answers as coherent and found that ChatGPT could provide bibliographic resources they could not find either within their standardized literature search or in open sources.

There are several limitations to our study. The sources of information used by language models like ChatGPT are vast and diverse, encompassing a wide range of data, including scientific studies, articles, books, and online sources. On the contrary, the sources used in MAs are usually more limited and specific, focusing on peer-reviewed studies and controlled trials that meet certain inclusion criteria, and it is so far impossible to confirm that the results of MAs are more reliable than those of an AI. Another major limitation in the interpretation of our data is that it is unknown to what extent, among the variety of sources analyzed by ChatGPT, the results and conclusions of the investigated MAs/NMAs have been used, especially when considering that GPT-3.5 is a series of models that was trained on a blend of text and code from before Q4 2021 [10,11]. Due to the potential overlap between the investigated MAs/NMAs, it is difficult to conclude whether ChatGPT was able to summarize these studies or simply output the information it was trained on.

While MAs and NMAs are usually regarded as one of the best tools to compare drug efficacy, they can be affected by a number of biases mainly because they are generated by humans. Besides, collecting data and evaluating the results of MAs/NMAs is often a long and arduous process. It is then quite disconcerting to receive an AI-based output in seconds that would take a human team years to develop and whose answers are more or less comparable to the conclusions of MAs/NMAs regarding the ranking of drug efficacy in the treatment of moderate-to-severe plaque psoriasis. However, at this point, it would be hazardous to rely solely on an AI to gather information since some mistakes from ChatGPT were found in this study, such as wrongly attributing certain drug characteristics or sometimes providing different rankings in terms of drug safety. Moreover, MAs/NMAs are scientific methods using statistical analyses, whereas an AI can be continuously trained to avoid this kind of mistake, and a language model such as ChatGPT could have the potential to be a fast and complementary method for processing the large volumes of data necessary to generate or verify the results of MAs/NMAs. However, since ChatGPT has so far had limitations in providing an accurate and complete overview of the available evidence, this technology should be applied under rigorous human supervision and control. The potential of other AI techniques or other pre-trained language models, such as BERT (bidirectional encoder representations from transformers) to simplify the process of summarizing data from the medical domain requires further investigation.

## Figures and Tables

**Table 1:** Efficacy and safety outcomes and main conclusions of the included studies from 2021–2023 and ChatGPT’s outputs.

Included Study (2021–2023)	Type of Study	Evaluated Molecules	Efficacy and Safety Outcomes (MA)	Most Effective Therapies (Meta-Analyses)	Tolerance (MA)	Most Effective Therapies (ChatGPT)	Tolerance (ChatGPT)
Pan et al., 2021 [19]	NMA	ADA, INF, SEC	Week 12/16/24 PASI 50/75/90	The efficacy of SEC is well demonstrated through NMA.	NA	The ranking of these biologics is SEC > ADA > INF.	NA
Fahrbach et al., 2021 [20]	NMA	ACT, ADA, APR, BRO, CSA, CZP, DMF, ETN, GUS, INF, IXE, MTX, RIS, SEC, TIL, UST	Week 10/16 PASI 50/75/90/100	IL inhibitors are likely the best short-term treatment choices.	NA	The short-term efficacy of treatments is: IXE > SEC > GUS > UST > ADA > ETA > INF > RIS > BRO > TIL > CZP > APR > DMF > MTX > CSA > ACT	NA
Shear et al., 2021 [21]	NMA	ADA, APR, BRO, CZP, DMF, ETN, GUS, INF, IXE, *RIS*, SEC, TIL, UST	Week 12/16 Week 48/56 PASI 90 Any AE, any SAE, AEs leading to treatment discontinuation	RIS had the most favorable benefit-risk profile in the long term.	Anti-IL 23 agents were associated with low rates of safety events.	Inconclusive.	Inconclusive.
He et al., 2021 [22]	NMA	ADA, ALE, APR, BAR, BRI, BRO, CZP, ETN, GUS, INF, IXE, MTX, RIS, SEC, TIL, TOF, UST	Week 12/16/24 PASI 75/90	RIS and IXE showed superiority for PASI 75 and PASI 90.	NA	Inconclusive.	NA
Almohideb M., 2021 [23]	MA	INF, RIS	Week 10 PASI 75/90 PGA Any AE, SAE	RIS is preferred over INF, and is significantly more effective.	RIS is significantly safer than INF.	RIS is more effective than INF at maintaining skin clearance after treatment has been discontinued.	RIS and INF have similar safety profiles.
Armstrong, Soliman, Betts et al., 2021 [24]	NMA	ADA, APR, BRO, CZP, DMF, ETN, GUS, INF, IXE, RIS, SEC, TIL, UST	Week 10/16 PASI 75/90/100 SUCRA	IXE, RIS, and BRO had the highest short-term efficacy.	NA	IXE, RIS, BRO, GUS, SEC, and TIL have the highest short-term efficacy.	NA
		ADA, BRO, ETN GUS, IXE, RIS, SEC, UST	Week 48/52 PASI 75/90/100 SUCRA	RIS had the highest long-term efficacy.		IXE, SEC, and UST have the highest long-term efficacy.	
Mrowietz et al., 2021 [25]	NMA	ADA, BRO, CZP, ETN, GUS, INF, IXE, RIS, SEC, TIL, UST	Week 10/16 Absolute PASI values ≤ 1, 2, 3, 5	The most effective treatments were BRO and IXE, followed by GUS and RIS.	NA	Inconclusive.	NA
Ravasio et al., 2021 [26]	NMA	BRO, GUS, IXE, SEC, RIS, TIL, UST	Week 8/24 PASI 90 NNT	IXE is the most effective option (NNT).	NA	IXE, SEC, UST, and GUS are highly effective. TIL is moderately effective. BRO is an effective treatment, although its safety profile is still being evaluated. The efficacy of RIS is still being studied.	NA
Torres et al., 2021 [27]	NMA	ADA, BRI, BRO, CZP, ETN, GUS, IXE, RIS, SEC, TIL, UST	Week 10/16 PASI 100 Week 24 ACR 70	SEC and IXE were the treatments with the highest probability of reaching both PASI100 and ACR70 outcomes.	NA	In reaching PASI100 and ACR70 outcomes: IXE, SEC, GUS, and BRO are highly effective, UST and ADA are also effective, but to a slightly lesser extent, CZP and ETN are moderately effective, BRI and RIS are still being evaluated. TIL is effective in reaching PASI100 but its ability to reach ACR70 is still being evaluated.	NA
Fu and Guo, 2022 [12]	MA	ADA, GUS	Week 16/20 PASI 75/90/100 PGA 0/1 DLQI 0/1	GUS showed better efficacy than ADA.	NA	GUS is more effective than ADA.	GUS and ADA have similar safety profile.
Yu et al., 2022 [13]	MA	RIS, UST	Week 16 Week 52 PASI 75/90/100 PGA 0/1 AE, SAE	RIS was more effective than UST.	AE of both RIS and UST were similar.	RIS was superior in terms of achieving clear or almost clear skin (PASI 90 and PASI 100).	Both RIS and UST are well-tolerated, with a similar incidence of AE.
Armstrong, Fahrbach et al., 2022 [14]	NMA	ACT, ADA, APR, BIM, BRO, CSA, CZP, DMF, ETN, GUS, INF, IXE, MTX, RIS, SEC, TIL, UST	Week 16/20 PASI 90/100 NNT	IL-17 and IL-23 inhibitors were highly effective in achieving short-term improvement, especially BIM.	NA	BIM and RIS may have a faster onset of action and greater efficacy compared to the other drugs.	NA
Leonardi et al., 2022 [15]	NMA	ADA, BRO, CZP, ETN, GUS, INF, IXE, RIS, SEC, TIL, UST	Week 12/16 PASI 90/100 NNT	BRO and IXE had the lowest NNTs for achieving PASI responses at early time points.	NA	Some biologics that have shown to have a relatively fast onset of action include: IXE, SEC, GUS, TIL.	NA
		ADA, BRO, CZP, ETN, GUS, INF, IXE, RIS, SEC, TIL, UST	Week 48/52 PASI 90/100 NNT	BRO and IXE were not significantly different than RIS and GUS after 48/52 weeks.		These biologics have demonstrated high levels of long-term efficacy: IXE, SEC, GUS, TIL, UST, ADA, INF.	
Blauvelt et al., 2022 [16]	NMA	ADA, BRO, CZP, ETN, GUS, INF, IXE, RIS, SEC, UST	Week 52 PASI 90/100 AUC	IXE and RIS provided the greatest cumulative clinical benefits over 1 year.	NA	These biologics have demonstrated high levels of efficacy after 1 year of use: IXE, SEC, GUS, TIL, UST, and INF.	NA
Yasmeen et al., 2022 [17]	NMA	ADA, APR, BRO, CZP, ETN, GUS, INF, IXE, RIS, SEC, UST	Week 52 PASI 75/90/100	IL-17 and IL-23 inhibitors outperformed other biologics after 1 year. RIS had a higher probability of achieving PASI outcomes over all other biologics, except BRO and GUS (no significant difference).	NA	These treatments have demonstrated high levels of efficacy after 1 year of use: IXE, SEC, GUS, UST, and INF.	NA
Armstrong, Soliman, Betts et al., 2022 [18]	NMA	ADA, BIM, BRO, ETN, GUS, IXE, RIS, SEC, UST	Week 48–56 PASI 75/90/100 SUCRA Any AE, any SAE, and AEs leading to treatment discontinuation	RIS was associated with the most favorable long-term benefit-risk profile. IXE and BIM also had favorable efficacy profiles.	IXE and BIM had lower rankings for safety outcomes.	Inconclusive.	Inconclusive.

*ACR*, American College of Rheumatology; *ACT*, acitretin; *ADA*, adalimumab; *AE*, adverse events; *ALE*, alefacept; *APR*, apremilast; *AUC*, area under the curve; *BAR,* baricitinib; *BIM*, bimekizumab; *BRI*, briakinumab; *BRO*, brodalumab; *CSA*, ciclosporin; *CZP*, certolizumab pegol; *DMF*, dimethyl fumarate; *ETN*, etanercept; *GUS*, guselkumab; *IL,* interleukin; *INF*, infliximab; *IXE*, ixekizumab; *MA*, meta-analysis; *MTX*, methotrexate; *NA,* Not applicable; *NMA*, network meta-analysis; *NNT*, number needed to treat; *PASI*, psoriasis area severity index; *RIS*, risankizumab; *SAE*, serious adverse events; *SEC*, secukinumab; *SUCRA*, surface under the cumulative ranking curve; *TIL*, tildrakizumab; *TOF*, tofacitinib; *UST*, ustekinumab.

**Table 2:** Summary of efficacy and safety comparisons between the conclusions of the included studies and ChatGPT’s answers.

Study	Type of Analysis	Efficacy Comparison	Safety Comparison
Fu and Guo, 2022 [12]	MA	=	NA
Yu et al., 2022 [13]	MA	=	=
Armstrong, Fahrbach et al., 2022 [14]	NMA	=	NA
Leonardi et al., 2022 [15]	NMA	±	NA
Blauvelt et al., 2022 [16]	NMA	±	NA
Yasmeen et al., 2022 [17]	NMA	±	NA
Armstrong, Soliman, Betts et al., 2022 [18]	NMA	IA	IA
Pan et al., 2021 [19]	NMA	=	NA
Fahrbach et al., 2021 [20]	NMA	±	NA
Shear et al., 2021 [21]	NMA	IA	IA
He et al., 2021 [22]	NMA	IA	NA
Almohideb M., 2021 [23]	MA	=	≠
Almohideb M., 2021 [23]	NMA	±	NA
Mrowietz et al., 2021 [25]	NMA	IA	NA
Ravasio et al., 2021 [26]	NMA	±	NA
Torres et al., 2021 [27]	NMA	±	NA

*IA*, inconclusive answer; *MA*, meta-analysis; *NA*, not applicable; *NMA*, network meta-analysis; =, identical result; ±, acceptable result; ≠, different result.

## Data Availability

The authors confirm that they have full access to all the data in the study and take responsibility for the integrity of the data and the accuracy of the data analysis. Supplementary data are available in the supplementary files.

## Supplementary Materials

The following supporting information can be downloaded at: https://www.mdpi.com/article/10.3390/jcm12165410/s1.

## References

[1] Sackett D.L. (2000). Evidence-Based Medicine: How to Practice and Teach EBM.

[2] Conn V.S., Ruppar T.M., Phillips L.J., Chase J.-A.D. (2012). Using meta-analyses for comparative effectiveness research. Nurs. Outlook.

[3] Cheung M.W.-L., Vijayakumar R. (2016). A Guide to Conducting a Meta-Analysis. Neuropsychol. Rev..

[4] Higgins J.P.T., Hoboken N.J., Cochrane Collaboration (2019). Cochrane Handbook for Systematic Reviews of Interventions.

[5] Tsafnat G., Glasziou P., Choong M.K., Dunn A., Galgani F., Coiera E. (2014). Systematic review automation technologies. Syst. Rev..

[6] Shojania K.G., Sampson M., Ansari M.T., Ji J., Doucette S., Moher D. (2007). How Quickly Do Systematic Reviews Go Out of Date? A Survival Analysis. Ann. Intern. Med..

[7] van de Schoot R., de Bruin J., Schram R., Zahedi P., de Boer J., Weijdema F., Kramer B., Huijts M., Hoogerwerf M., Ferdinands G. (2021). An open source machine learning framework for efficient and transparent systematic reviews. Nat. Mach. Intell..

[8] Renders J.-M., Simonart T. (2009). Role of artificial neural networks in dermatology. Dermatology.

[9] Michelson M., Chow T., A Martin N., Ross M., Ying A.T.Q., Minton S. (2020). Artificial Intelligence for Rapid Meta-Analysis: Case Study on Ocular Toxicity of Hydroxychloroquine. J. Med. Internet Res..

[10] OpenAI ChatGPT: Optimizing Language Models for Dialogue. https://openai.com/blog/chatgpt/.

[11] Browne R. All You Need to Know about ChatGPT, the A.I. Chatbot That’s Got the World Talking and Tech Giants Clashing. https://www.cnbc.com/2023/02/08/what-is-chatgpt-viral-ai-chatbot-at-heart-of-microsoft-google-fight.html.

[12] Fu H., Guo J. (2022). Efficacy of guselkumab compared with adalimumab for psoriasis: A meta-analysis of randomized controlled studies. Adv. Dermatol. Allergol..

[13] Yu Q., Ge X., Jing M., Mi X., Guo J., Xiao M., Lei Q., Chen M. (2022). A Systematic Review with Meta-Analysis of Comparative Efficacy and Safety of Risankizumab and Ustekinumab for Psoriasis Treatment. J. Immunol. Res..

[14] Armstrong A., Fahrbach K., Leonardi C., Augustin M., Neupane B., Kazmierska P., Betts M., Freitag A., Kiri S., Taieb V. (2022). Efficacy of Bimekizumab and Other Biologics in Moderate to Severe Plaque Psoriasis: A Systematic Literature Review and a Network Meta-Analysis. Dermatol. Ther..

[15] Leonardi C.L., See K., Burge R., Sun Z., Zhang Y., Mallbris L., Garrelts A., Warren R.B. (2022). Number Needed to Treat Network Meta-Analysis to Compare Biologic Drugs for Moderate-to-Severe Psoriasis. Adv. Ther..

[16] Blauvelt A., Gooderham M., Griffiths C.E.M., Armstrong A.W., Zhu B., Burge R., Gallo G., Guo J., Garrelts A., Lebwohl M. (2022). Cumulative Clinical Benefits of Biologics in the Treatment of Patients with Moderate-to-Severe Psoriasis over 1 Year: A Network Meta-Analysis. Dermatol. Ther..

[17] Yasmeen N., Sawyer L.M., Malottki K., Levin L., Apol E.D., Jemec G.B. (2020). Targeted therapies for patients with moderate-to-severe psoriasis: A systematic review and network meta-analysis of PASI response at 1 year. J. Dermatol. Treat..

[18] Armstrong A.W., Soliman A.M., Betts K.A., Wang Y., Gao Y., Stakias V., Puig L. (2021). Long-Term Benefit–Risk Profiles of Treatments for Moderate-to-Severe Plaque Psoriasis: A Network Meta-Analysis. Dermatol. Ther..

[19] Pan R., Wang X., Shu M., Das J., Kalra M., Wang Z. (2021). Comparative efficacy of secukinumab against adalimumab and infliximab in patients with moderate-to-severe plaque psoriasis. Chin. Med. J..

[20] Fahrbach K., Sarri G., Phillippo D.M., Neupane B., Martel S.E., Kiri S., Reich K. (2021). Short-Term Efficacy of Biologic Therapies in Moderate-to-Severe Plaque Psoriasis: A Systematic Literature Review and an Enhanced Multinomial Network Meta-Analysis. Dermatol. Ther..

[21] Shear N.H., Betts K.A., Soliman A.M., Joshi A., Wang Y., Zhao J., Gisondi P., Sinvhal R., Armstrong A.W. (2021). Comparative safety and benefit-risk profile of biologics and oral treatment for moderate-to-severe plaque psoriasis: A network meta-analysis of clinical trial data. J. Am. Acad. Dermatol..

[22] He H., Wu W., Zhang Y., Zhang M., Sun N., Zhao L., Wang X. (2021). Model-Based Meta-Analysis in Psoriasis: A Quantitative Comparison of Biologics and Small Targeted Molecules. Front. Pharmacol..

[23] Almohideb M. (2021). Safety and Efficacy of Risankizumab and Infliximab in the Treatment of Plaque Psoriasis: Results From a Direct and Indirect Meta-Analysis. Cureus.

[24] Armstrong A.W., Soliman A.M., Betts K.A., Wang Y., Gao Y., Puig L., Augustin M. (2021). Comparative Efficacy and Relative Ranking of Biologics and Oral Therapies for Moderate-to-Severe Plaque Psoriasis: A Network Meta-analysis. Dermatol. Ther..

[25] Mrowietz U., Warren R., Leonardi C., Saure D., Petto H., Hartz S., Dossenbach M., Reich K. (2021). Network meta-analysis of biologic treatments for psoriasis using absolute Psoriasis Area and Severity Index values ≤1, 2, 3 or 5 derived from a statistical conversion method. J. Eur. Acad. Dermatol. Venereol..

[26] Ravasio R., Costanzo A., Antonelli S., Maiorino A., Losi S. (2021). Number needed to treat for interleukin inhibitors approved for the treatment of moderate-to-severe plaque psoriasis in Italy. Glob. Reg. Health Technol. Assess..

[27] Torres T., Barcelos A., Filipe P., Fonseca J.E. (2021). A Systematic Review with Network Meta-Analysis of the Available Biologic Therapies for Psoriatic Disease Domains. Front. Med..

[28] Singh S., Singh S., Thangaswamy A., Thangaraju P., Varthya S.B. (2020). Efficacy and safety of Risankizumab in moderate to severe psoriasis: A systematic review and meta-analysis. Dermatol. Ther..

[29] Zhang L., Guo L., Wang L., Jiang X. (2022). The efficacy and safety of tofacitinib, peficitinib, solcitinib, baricitinib, abrocitinib and deucravacitinib in plaque psoriasis—A network meta-analysis. J. Eur. Acad. Dermatol. Venereol..

[30] Xu S., Gao X., Deng J., Yang J., Pan F. (2020). Comparative efficacy and safety of biologics in moderate to severe plaque psoriasis: A multiple-treatments meta-analysis. JDDG J. Dtsch. Dermatol. Ges..

[31] Puig L. (2021). Meta-analysis and Indirect Comparisons: On Methods, Paradigms, and Biologic Treatments for Psoriasis. Actas Dermosifiliogr. (Engl. Ed.).

[32] Kang Q., Chen J.-S., Yang H. (2022). Efficacy and safety profile of phosphodiesterase 4 inhibitor in the treatment of psoriasis: A systematic review and meta-analysis of randomized controlled trials. Front. Immunol..

[33] Sarabia S., Ranjith B., Koppikar S., Wijeratne D.T. (2022). Efficacy and safety of JAK inhibitors in the treatment of psoriasis and psoriatic arthritis: A systematic review and meta-analysis. BMC Rheumatol..

[34] Aljefri Y.E., Ghaddaf A.A., Alkhunani T.A., Alkhamisi T.A., Alahmadi R.A., Alamri A.M., Alraddadi A.A. (2022). Efficacy and safety of apremilast monotherapy in moderate-to-severe plaque psoriasis: A systematic review and meta-analysis. Dermatol. Ther..

[35] Zhu T., Ma L. (2022). Meta-Analysis of the Efficacy and Safety of Interleukin-23-Targeted Drugs in the Treatment of Moderate-to-Severe Psoriasis. Contrast Media Mol. Imaging.

[36] Song G.G., Lee Y.H. (2021). Relative efficacy and safety of tofacitinib for treating psoriasis: A Bayesian network meta-analysis of randomized controlled trials. Int. J. Clin. Pharmacol. Ther..

[37] Sbidian E., Chaimani A., Garcia-Doval I., Doney L., Dressler C., Hua C., Hughes C., Naldi L., Afach S., Le Cleach L. (2022). Systemic pharmacological treatments for chronic plaque psoriasis: A network meta-analysis. Cochrane Database Syst. Rev..

[38] Sbidian E., Chaimani A., Garcia-Doval I., Doney L., Dressler C., Hua C., Hughes C., Naldi L., Afach S., Le Cleach L. (2021). Systemic pharmacological treatments for chronic plaque psoriasis: A network meta-analysis. Cochrane Database Syst. Rev..

[39] Smith C.H., Mahil S.K., Yiu Z.Z., Bale T., Burden A.D., Coates L.C., McGuire A., Murphy R., Owen C.M., Parslew R. (2021). Quantitative Evaluation of Biologic Therapy Options for Psoriasis: A Systematic Review and Network Meta-Analysis–Correction. J. Investig. Dermatol..

[40] LeLorier J., Grégoire G., Benhaddad A., Lapierre J., Derderian F. (1997). Discrepancies between Meta-Analyses and Subsequent Large Randomized, Controlled Trials. N. Engl. J. Med..

[41] Tonin F.S., Araujo A.G., Fachi M.M., Ferreira V.L., Pontarolo R., Fernandez-Llimos F. (2021). Lag times in the publication of network meta-analyses: A survey. BMJ Open.

[42] Paez A. (2017). Gray literature: An important resource in systematic reviews. J. Evid. Based Med..

[43] Zhou J., Müller H., Holzinger A., Chen F. Ethical ChatGPT: Concerns, Challenges, and Commandments. https://arxiv.org/pdf/2305.10646.pdf.

[44] Anghelescu A., Firan F.C., Onose G., Munteanu C., Trandafir A.-I., Ciobanu I., Gheorghița Ș., Ciobanu V. (2023). PRISMA Systematic Literature Review, including with Meta-Analysis vs. Chatbot/GPT (AI) regarding Current Scientific Data on the Main Effects of the Calf Blood Deproteinized Hemoderivative Medicine (Actovegin) in Ischemic Stroke. Biomedicines.

