# Supplementary material for: Comparing Meta-Analyses with ChatGPT in the Evaluation of the Effectiveness and Tolerance of Systemic Therapies in Moderate-to-Severe Plaque Psoriasis
Source: J Clin Med. 2023 Aug 20;12(16):5410. doi: 10.3390/jcm12165410 (PMC10455399; doi:10.3390/jcm12165410)
Supplement: Supplementary file 1 [file jcm-12-05410-s001.zip › jcm-2555260-SI.pdf]

Supplemental material

1. Efficacy of **guselkumab** compared with **adalimu2mab** for psoriasis: a meta-analysis of randomized controlled studies. Fu H, Guo J. Postepy Dermatol Alergol. 2022 Oct;39(5):953-958.

Conclusion of the MA: Guselkumab showed better efficacy than adalimumab for psoriasis.
Query: Compare the efficacy of guselkumab and adalimumab for psoriasis.
ChatGPT output: Guselkumab and adalimumab are both biologic medications that are used to treat moderate to severe psoriasis. Both medications work by blocking the action of a specific protein in the body called tumor necrosis factor alpha (TNF-alpha), which plays a role in the development of psoriasis. Guselkumab is a human monoclonal antibody that targets the interleukin (IL)-23 pathway, while adalimumab is a human monoclonal antibody that targets TNF-alpha. Clinical trials have shown that guselkumab is more effective than adalimumab in treating psoriasis. In one study, 80% of patients treated with guselkumab achieved a 75% improvement in their psoriasis symptoms, compared to 52% of patients treated with adalimumab. Additionally, in another study, 92% of patients treated with guselkumab achieved a 90% improvement in their psoriasis symptoms, compared to 69% of patients treated with adalimumab. It's worth to note, both medications have similar safety profile. It's important to consult a dermatologist for personalized treatment options.

2. A Systematic Review with Meta-Analysis of Comparative Efficacy and Safety of Risankizumab and Ustekinumab for **Psoriasis** Treatment. Yu Q, Ge X, Jing M, Mi X, Guo J, Xiao M, Lei Q, Chen M. J Immunol Res. 2022 Aug 18;2022:2802892.

Conclusion of the MA: Risankizumab was more effective than ustekinumab. Nevertheless, risankizumab and ustekinumab did not show significant difference in the incidence of adverse responses.
Query: Compare the efficacy and tolerance of risankizumab and ustekinumab for the treatment of psoriasis.
ChatGPT output: Risankizumab and ustekinumab are both biologic therapies that are used to treat moderate to severe psoriasis. Risankizumab is a monoclonal antibody that targets the interleukin-23 (IL-23) protein, which is thought to play a role in the development of psoriasis. Several studies have shown that risankizumab is highly effective in treating psoriasis, with a high percentage of patients achieving clear or almost clear skin. A phase 3 trial comparing risankizumab to ustekinumab found that risankizumab was superior in terms of achieving clear or almost clear skin, with a higher percentage of patients achieving PASI 90 and PASI 100. Ustekinumab, also a monoclonal antibody, targets the interleukin-12 and -23 proteins, which are involved in the immune response that leads to the development of psoriasis. Ustekinumab has been shown to be effective in treating psoriasis, with a high percentage of patients achieving clear or almost clear skin. However, in the phase 3 trial comparing risankizumab and ustekinumab, ustekinumab was found to be less effective than risankizumab in achieving clear or almost clear skin, with a lower percentage of patients achieving PASI 90 and PASI 100. In terms of tolerance, both risankizumab and ustekinumab have been found to be well-tolerated in clinical trials, with a similar incidence of adverse events reported for both medications. However, it is important to note that the best treatment option for an individual patient will depend on their specific circumstances, including the severity of their psoriasis and any other medical conditions they may have. It's best to consult a dermatologist for personalized advice.

3. Efficacy of **Bimekizumab** and Other **Biologics** in **Moderate** to **Severe Plaque Psoriasis**: A Systematic Literature Review and a Network Meta-Analysis. Armstrong A, Fahrbach K, Leonardi C, Augustin M, Neupane B, Kazmierska P, Betts M, Freitag

A, Kiri S, Taieb V, Slim M, Gomez NN, Warren RB. Dermatol Ther (Heidelb). 2022 Aug;12(8):1777-1792.

Conclusion of the MA: This analysis demonstrated that IL-17 and IL-23 inhibitors were highly effective in achieving short-term improvement among patients with moderate to severe psoriasis. Patients receiving bimekizumab were significantly more likely to achieve PASI 90 or PASI 100 within 10–16 weeks of the first injection than all other biologics.

Query: Compare the short-term efficacy of bimekizumab, risankizumab, ixekizumab, brodalumab, guselkumab and secukinumab for moderate to severe plaque psoriasis.

ChatGPT output 1: The short-term efficacy of these biologic drugs for severe plaque psoriasis has been studied and shown to be effective in clinical trials. However, specific comparison of efficacy between the drugs can vary based on the study design and population. It is important to consult a healthcare professional for personalized treatment recommendations.

ChatGPT output 2: All of the drugs listed (bimekizumab, risankizumab, ixekizumab, brodalumab, guselkumab, and secukinumab) are biologic medications used to treat moderate to severe plaque psoriasis. In clinical trials, these drugs have shown similar short-term efficacy in terms of clearing or improving skin plaques and reducing psoriasis symptoms. However, some studies suggest that bimekizumab and risankizumab may have a faster onset of action and greater efficacy compared to the other drugs. It is important to note that individual responses to these medications may vary, and the best choice for a particular patient depends on factors such as their specific symptoms, overall health, and previous treatment history. Consultation with a dermatologist or rheumatologist is recommended for proper evaluation and determination of the most appropriate treatment for each individual case.

4. <u>Number Needed to Treat Network Meta-Analysis to Compare Biologic Drugs for Moderate-to-Severe Psoriasis.</u> Leonardi C et al. Adv Ther 2022 May;39(5):2256-2269.

Conclusion of the MA: Brodalumab and ixekizumab had the lowest NNTs for achieving PASI responses at early time points and were not significantly different than risankizumab and guselkumab after 48/52 weeks.

Query: Which biologics have the lowest number needed to treat for achieving PASI responses at early time points?

ChatGPT output: The number needed to treat (NNT) is a measure of the effectiveness of a treatment, and varies depending on the study and population. Studies have shown that some biologic drugs, such as risankizumab, have a lower NNT compared to others for achieving PASI (Psoriasis Area and Severity Index) responses at early time points in patients with severe plaque psoriasis. However, it's important to note that individual response to treatment may vary and the NNT can change based on different study populations and designs. A healthcare professional should be consulted for personalized treatment recommendations.

Query: Which biologics among brodalumab, ixekizumab, secukinumab, guselkumab, risankizumab, tildrakizumab, ustekinumab, adalimumab, certolizumab pegol, etanercept and infliximab have the lowest number needed to treat for achieving PASI responses at early time points?

ChatGPT output: It is difficult to determine which biologic has the lowest number needed to treat (NNT) for achieving PASI responses at early time points, as NNT may vary based on the study population, disease severity, and other factors. However, some biologics, such as secukinumab, ixekizumab, and guselkumab, have been shown to have high NNT in clinical trials. It is recommended to consult with a healthcare provider for personalized treatment recommendations.

Query: Compare these biologics in terms of speed of efficacy in psoriasis treatment: brodalumab, ixekizumab, secukinumab, guselkumab, risankizumab, tildrakizumab, ustekinumab, adalimumab, certolizumab pegol, etanercept and infliximab.

ChatGPT output: For the treatment of psoriasis, some biologics may show improvement in as little as 4-12 weeks, while others may take longer. The speed of efficacy can vary and depend on several factors such as the severity of the condition and the individual patient's response. Some biologics that have shown to have a relatively fast onset of action in psoriasis treatment include: Ixekizumab, Secukinumab, Guselkumab, Tildrakizumab. It's important to note that individual results can vary and that it's recommended to consult a healthcare professional for specific treatment recommendations and for monitoring the effectiveness of the chosen treatment.

Query: Compare these biologics in terms of long-term efficacy in psoriasis treatment: brodalumab, ixekizumab, secukinumab, guselkumab, risankizumab, tildrakizumab, ustekinumab, adalimumab, certolizumab pegol, etanercept and infliximab.

ChatGPT output: Long-term efficacy of biologics in psoriasis treatment varies and can be influenced by factors such as the patient's individual response and severity of psoriasis. However, in general, the following biologics have demonstrated high levels of long-term efficacy in clinical trials and real-world studies: Ixekizumab, Secukinumab, Guselkumab, Tildrakizumab, Ustekinumab, Adalimumab, Infliximab. Other biologics such as brodalumab, risankizumab, certolizumab pegol, and etanercept have also demonstrated efficacy in treating psoriasis, but to a lesser extent compared to the above drugs. It's important to note that long-term efficacy can also depend on the patient's ability to continue taking the medication as directed and manage any side effects that may occur. Additionally, treatment plans may involve switching between biologics based on individual response and efficacy.

5. Cumulative Clinical Benefits of Biologics in the Treatment of Patients with Moderate-to-Severe Psoriasis over 1 Year: a Network Meta-Analysis. Blauvelt A et al. Dermatol Ther (Heidelb) 2022 Mar;12(3):727-740.

Conclusion of the MA: For complete and almost-complete skin clearance, ixekizumab and risankizumab provided the greatest cumulative clinical benefits over 1 year.

Query: Which biologic treatment provides the greatest cumulative clinical benefits over 1 year for complete and almost-complete skin clearance in psoriasis?

ChatGPT output: Studies have shown that several biologic drugs, including bimekizumab, risankizumab, ixekizumab, brodalumab, guselkumab, and secukinumab, can provide significant cumulative clinical benefits for complete and almost-complete skin clearance in psoriasis over a period of 1 year. The greatest cumulative clinical benefits in terms of skin clearance can vary based on individual patient characteristics, disease severity, and response to treatment. It is recommended to consult a healthcare professional for personalized treatment recommendations.

Query: Which biologic among ixekizumab, brodalumab, risankizumab, guselkumab, secukinumab, infliximab, ustekinumab, certolizumab pegol, adalimumab and etanercept provides the greatest cumulative clinical benefits over 1 year for complete and almost-complete skin clearance in psoriasis?

ChatGPT output: It is difficult to determine which biologic provides the greatest cumulative clinical benefits over 1 year for complete and almost-complete skin clearance in psoriasis, as this may vary based on the study population, disease severity, and other factors. However, some biologics, such as ixekizumab, secukinumab, and guselkumab, have been shown to provide high levels of skin clearance in clinical trials. It is recommended to consult with a healthcare provider for personalized treatment recommendations.

Query: Compare these biologics: ixekizumab, brodalumab, risankizumab, guselkumab, secukinumab, infliximab, ustekinumab, certolizumab pegol, adalimumab and etanercept, which one has the most efficacy in psoriasis treatment after 1 year?

ChatGPT output: The efficacy of biologics in psoriasis treatment can vary between patients and is dependent on individual factors such as disease severity and response to treatment. However, based on clinical trial data and real-world evidence, the following biologics have demonstrated high levels of efficacy in treating psoriasis after 1 year of use: Ixekizumab, Secukinumab, Guselkumab, Tildrakizumab, Ustekinumab, Infliximab. These biologics have shown high response rates and sustained improvement in skin clearance and quality of life in patients with moderate to severe psoriasis after 1 year of treatment. Other biologics such as brodalumab, risankizumab, certolizumab pegol, adalimumab, and etanercept have also demonstrated efficacy in treating psoriasis, but to a lesser extent compared to the above drugs. It is important to note that individual response to biologics may vary, and treatment plans may involve switching between biologics based on individual response and efficacy. A healthcare professional should be consulted to determine the most effective treatment plan for an individual patient.

6. Targeted therapies for patients with moderate-to-severe psoriasis: a systematic review and network meta-analysis of PASI response at 1 year. Yasmeen N et al. J Dermatolog Treat 2022 Feb;33(1):204-218.

Conclusion of the MA: This NMA demonstrated that evaluated IL-17 and IL-23 inhibitors outperformed other biological therapies after 1 year. Risankizumab had a higher probability of achieving PASI outcomes than all other biologics, except brodalumab and guselkumab, where no significant difference could be concluded.
Query: In psoriasis treatment, which biologics outperform the others after 1 year?
ChatGPT output: The effectiveness of biologic drugs for treating psoriasis can vary depending on individual patient characteristics, disease severity, and response to treatment. Studies have shown that several biologic drugs, including bimekizumab, risankizumab, ixekizumab, brodalumab, guselkumab, and secukinumab, can provide significant clinical benefits for the treatment of psoriasis over a period of 1 year. It's difficult to say which biologic outperforms the others after 1 year as it can depend on the patient population, study design, and criteria for evaluating outcomes. A healthcare professional should be consulted for personalized treatment recommendations.
Query: Which biologic treatment for psoriasis has a higher probability of achieving PASI outcomes?
ChatGPT output: The probability of achieving PASI (Psoriasis Area and Severity Index) outcomes with biologic treatment for psoriasis can vary based on individual patient characteristics, disease severity, and response to treatment. Studies have shown that several biologic drugs, including bimekizumab, risankizumab, ixekizumab, brodalumab, guselkumab, and secukinumab, can effectively achieve PASI outcomes in patients with psoriasis. It's difficult to determine which biologic treatment has a higher probability of achieving PASI outcomes as the results can vary based on the study population, design, and criteria for evaluating outcomes. It is recommended to consult a healthcare professional for personalized treatment recommendations.
Query: For moderate to severe psoriasis, which treatment among brodalumab, ixekizumab, secukinumab, guselkumab, risankizumab, ustekinumab, adalimumab, certolizumab pegol, etanercept, infliximab and apremilast outperforms the others after 1 year and has the highest probability of achieving PASI outcomes?
ChatGPT output: It is difficult to determine which treatment among brodalumab, ixekizumab, secukinumab, guselkumab, risankizumab, ustekinumab, adalimumab, certolizumab pegol, etanercept, infliximab, and apremilast outperforms the others after 1 year and has the highest probability of achieving PASI outcomes for moderate to severe psoriasis, as this may vary based on the study population, disease severity, and other factors. Clinical trials have shown that some

biologics, such as ixekizumab, secukinumab, and guselkumab, and some small molecule treatments, such as apremilast, have demonstrated high levels of skin clearance and PASI outcomes. It is recommended to consult with a healthcare provider for personalized treatment recommendations.

Query: For moderate to severe psoriasis, compare these treatments efficacy after 1 year: brodalumab, ixekizumab, secukinumab, guselkumab, risankizumab, ustekinumab, adalimumab, certolizumab pegol, etanercept, infliximab and apremilast.

ChatGPT output: For moderate to severe psoriasis, the efficacy of different treatments can vary between patients and is dependent on individual factors such as disease severity and response to treatment. However, based on clinical trial data and real-world evidence, the following treatments have demonstrated high levels of efficacy in treating moderate to severe psoriasis after 1 year of use: Ixekizumab, Secukinumab, Guselkumab, Ustekinumab, Infliximab. These treatments have shown high response rates and sustained improvement in skin clearance and quality of life in patients with moderate to severe psoriasis after 1 year of treatment. Other treatments such as brodalumab, risankizumab, adalimumab, certolizumab pegol, etanercept, and apremilast have also demonstrated efficacy in treating psoriasis, but to a lesser extent compared to the above drugs. It is important to note that individual response to treatments may vary, and treatment plans may involve switching between treatments based on individual response and efficacy. A healthcare professional should be consulted to determine the most effective treatment plan for an individual patient.

7. Long-Term Benefit-Risk Profiles of Treatments for Moderate-to-Severe Plaque Psoriasis: A Network Meta-Analysis. Armstrong AW, Soliman AM, Betts KA, Wang Y, Gao Y, Stakias V, Puig L. Dermatol Ther (Heidelb) 2022 Jan;12(1):167-184.

Conclusion of the MA: Risankizumab was associated with the most favorable long-term benefit-risk profile for the treatment of moderate-to-severe plaque psoriasis. Although ixekizumab and bimekizumab had favorable efficacy profiles, both treatments had lower rankings for safety outcomes.

Query: Which biologic is associated with the most favorable long-term benefit-risk profile for the treatment of moderate-to-severe plaque psoriasis?

ChatGPT output: The long-term benefit-risk profile of biologic drugs for the treatment of moderate-to-severe plaque psoriasis can vary depending on individual patient characteristics and response to treatment. Studies have shown that several biologic drugs, including bimekizumab, risankizumab, ixekizumab, brodalumab, guselkumab, and secukinumab, can effectively treat moderate-to-severe plaque psoriasis with a favorable benefit-risk profile. It's difficult to determine which biologic is associated with the most favorable long-term benefit-risk profile as the results can vary based on the study population, design, and criteria for evaluating outcomes. It is recommended to consult a healthcare professional for personalized treatment recommendations and to discuss the potential benefits and risks of each treatment option.

Query: Rank these biologics: bimekizumab, brodalumab, ixekizumab, secukinumab, guselkumab, risankizumab, ustekinumab, adalimumab and etanercept as those associated with the most favorable long-term benefit-risk profile for the treatment of moderate-to-severe plaque psoriasis.

ChatGPT output: It is difficult to rank these biologics in terms of the most favorable long-term benefit-risk profile for the treatment of moderate-to-severe plaque psoriasis, as this may vary based on the study population, disease severity, and other factors. Some biologics, such as ixekizumab, secukinumab, and guselkumab, have demonstrated high levels of skin clearance and favorable benefit-risk profiles in clinical trials, while others, such as adalimumab and

etanercept, have a longer history of use and established safety profiles. It is recommended to consult with a healthcare provider for personalized treatment recommendations.

8. Comparative efficacy of secukinumab against adalimumab and infliximab in patients with moderate-to-severe plaque psoriasis. Pan R, Wang X, Shu M, Das J, Kalra M, Wang Z. Chin Med J (Engl) 2021 Dec 9;135(1):11-19.

Conclusion of the MA: The efficacy of secukinumab in the treatment of patient populations with moderate-to-severe plaque psoriasis is well demonstrated through NMA.

Query: Rank the efficacy of secukinumab, adalimumab and infliximab in patients with moderate to severe psoriasis.

ChatGPT output 1: The efficacy of secukinumab, adalimumab, and infliximab for the treatment of moderate to severe psoriasis has been demonstrated in clinical trials. In terms of ranking the efficacy, it is difficult to make a direct comparison as the results can vary based on the study population, design, and criteria for evaluating outcomes. All three drugs have been shown to be effective in treating moderate to severe psoriasis, and the best option for a specific patient will depend on individual characteristics and response to treatment. It is recommended to consult a healthcare professional for personalized treatment recommendations and to discuss the potential benefits and risks of each treatment option.

ChatGPT output 2: The efficacy of biologic treatments for moderate to severe psoriasis can vary based on individual patient factors, but in general the following ranking of treatments is supported by clinical evidence: 1. Secukinumab, 2. Adalimumab, 3. Infliximab. It is important to note that the individual response to these treatments can vary greatly, and a different ranking may be observed in a specific patient population or study. A patient's response to a particular treatment should be determined through careful consideration of their individual medical history and in consultation with their healthcare provider.

9. Short-Term Efficacy of Biologic Therapies in Moderate-to-Severe Plaque Psoriasis: A Systematic Literature Review and an Enhanced Multinomial Network Meta-Analysis. Fahrbach K, Sarri G, Phillippo DM, Neupane B, Martel SE, Kiri S, Reich K. Dermatol Ther (Heidelb) 2021 Dec;11(6):1965-1998.

Conclusion of the MA: The results of this model confirmed that IL inhibitors are likely the best short-term treatment choices for improving all PASI levels.

Query: Rank the short-term efficacy of biologic therapies in moderate to severe plaque psoriasis.

ChatGPT output: The short-term efficacy of biologic therapies in moderate to severe plaque psoriasis can vary based on individual patient characteristics, disease severity, and response to treatment. Studies have shown that several biologic drugs, including bimekizumab, risankizumab, ixekizumab, brodalumab, guselkumab, secukinumab, adalimumab, and infliximab, can effectively treat moderate to severe plaque psoriasis with high levels of efficacy in the short term. It's difficult to rank the short-term efficacy of these drugs as the results can vary based on the study population, design, and criteria for evaluating outcomes. It is recommended to consult a healthcare professional for personalized treatment recommendations and to discuss the potential benefits and risks of each treatment option.

Query: Rank the short-term efficacy of these treatments in moderate to severe plaque psoriasis: brodalumab, ixekizumab, secukinumab, guselkumab, risankizumab, tildrakizumab, ustekinumab, adalimumab, certolizumab pegol, etanercept, infliximab, apremilast, dimethyl fumarate, methotrexate, cyclosporin and acitretin.

ChatGPT output 1: It is difficult to rank the short-term efficacy of these biologic and systemic therapies in moderate to severe plaque psoriasis, as this may vary based on the study population, disease severity, and other factors. However, some biologics, such as ixekizumab,

secukinumab, and guselkumab, and some small molecule treatments, such as apremilast, have demonstrated high levels of skin clearance in clinical trials. It is recommended to consult with a healthcare provider for personalized treatment recommendations.

ChatGPT output 2: The short-term efficacy of treatments for moderate to severe plaque psoriasis, ranked from highest to lowest, is as follows: 1. Ixekizumab 2. Secukinumab 3. Guselkumab 4. Ustekinumab 5. Adalimumab 6. Etanercept 7. Infliximab 8. Risankizumab 9. Brodalumab 10. Tildrakizumab 11. Certolizumab pegol 12. Apremilast 13. Dimethyl fumarate 14. Methotrexate 15. Cyclosporin 16. Acitretin. Note: This ranking is based on the available clinical trial data and can vary based on individual patient characteristics, disease severity, and other factors.

10. Comparative safety and benefit-risk profile of biologics and oral treatment for moderate-to-severe plaque **psoriasis**: A network meta-analysis of clinical trial data. Shear NH, Betts KA, Soliman AM, Joshi A, Wang Y, Zhao J, Gisondi P, Sinvhal R, Armstrong AW. J Am Acad Dermatol. 2021 Sep;85(3):572-581.

Conclusion of the MA: Anti-interleukin 23 agents were associated with low rates of safety events. Risankizumab had the most favorable benefit-risk profile in the long term.

Query: Rank the tolerance and benefit-risk profile in the long term of Risankizumab, Adalimumab, Apremilast, Brodalumab, Certolizumab pegol, Dimethyl fumarate, Etanercept, Guselkumab, Infliximab, Ixekizumab, Secukinumab, Tildrakizumab, Ustekinumab for the treatment of psoriasis.

ChatGPT output: It is not possible to rank the efficacy of these drugs as they are used to treat different medical conditions and each drug may work differently for each individual. The effectiveness of a particular drug also depends on various factors such as the severity of the condition being treated, the individual's medical history, and the presence of other medical conditions. It is important to consult a healthcare professional for the most appropriate treatment plan for your specific needs.

11. Model-Based Meta-Analysis in Psoriasis: A Quantitative Comparison of Biologics and Small Targeted Molecules. He H, Wu W, Zhang Y, Zhang M, Sun N, Zhao L, Wang X. Front Pharmacol 2021 Jul 1;12:586827.

Conclusion of the MA: This study provided a quantitative efficacy comparison of 17 systemic agents for psoriasis in term of efficacy only; safety was not considered. Risankizumab and ixekizumab showed superiority for both the two end points.

Query: Rank the efficacy of brodalumab, ixekizumab, secukinumab, guselkumab, risankizumab, tildrakizumab, ustekinumab, briakinumab, adalimumab, certolizumab pegol, etanercept, infliximab, apremilast, methotrexate, alefacept, tofacitinib and baricitinib in terms of PASI 75 and PASI 90 at week 12, 16 and 24.

ChatGPT output: Ranking the efficacy of the various biologic and non-biologic treatments in terms of PASI 75 (75% improvement in the Psoriasis Area and Severity Index) and PASI 90 (90% improvement in the Psoriasis Area and Severity Index) at week 12, 16, and 24 can vary based on the study population, design, and criteria for evaluating outcomes. Studies have shown that several of these treatments can effectively treat moderate to severe plaque psoriasis, with high levels of efficacy in terms of PASI 75 and PASI 90, including: Bimekizumab, Risankizumab, Ixekizumab, Brodalumab, Guselkumab, Secukinumab, Tildrakizumab, Ustekinumab, Briakinumab, Adalimumab, Certolizumab pegol, Etanercept, Infliximab. It is difficult to determine an exact ranking of efficacy as the results can vary based on the study population, design, and criteria for evaluating outcomes. Additionally, the best option for a specific patient will depend on individual characteristics and response to treatment. It is

recommended to consult a healthcare professional for personalized treatment recommendations and to discuss the potential benefits and risks of each treatment option.

Query: Rank the efficacy of brodalumab, ixekizumab, secukinumab, guselkumab, risankizumab, tildrakizumab, ustekinumab, briakinumab, adalimumab, certolizumab pegol, etanercept, infliximab, apremilast, methotrexate, alefacept, tofacitinib and baricitinib.

ChatGPT output: It's difficult to rank the efficacy of all these drugs as it largely depends on the individual patient and the condition being treated. However, some drugs are generally considered to be more effective than others for certain conditions. Here is a general overview of the efficacy of these drugs:

1. Infliximab, etanercept, secukinumab, ixekizumab, adalimumab, guselkumab, and risankizumab are biologic drugs that are highly effective in treating moderate-to-severe conditions such as psoriasis, rheumatoid arthritis, and ankylosing spondylitis.
2. Apremilast and tofacitinib are orally administered drugs that are highly effective in treating psoriasis and rheumatoid arthritis.
3. Baricitinib is an orally administered drug that is highly effective in treating rheumatoid arthritis.
4. Methotrexate, alefacept, and briakinumab are moderately effective in treating psoriasis and rheumatoid arthritis.
5. Certolizumab pegol and infliximab are moderately effective in treating Crohn's disease.
6. Tildrakizumab and ustekinumab are moderately effective in treating psoriasis.
7. The efficacy of brodalumab and certolizumab pegol in treating psoriasis is still being studied.

It is important to note that this is a general overview and that the efficacy of these drugs may vary between individuals and conditions. It is recommended to consult with a healthcare professional to determine the best treatment plan for your specific needs.

12. **Safety** and Efficacy of **Risankizumab** and **Infliximab** in the Treatment of Plaque Psoriasis: Results From a Direct and Indirect Meta-Analysis. Almohideb M. Cureus. 2021 Jun 27;13(6):e15963.

Conclusion of the MA: Risankizumab is preferred for the treatment of psoriasis than infliximab, and is significantly more effective and safe.

Query: Compare the safety and efficacy of infliximab and risankizumab in the treatment of plaque psoriasis.

ChatGPT output: Both infliximab and risankizumab are biologic medications used to treat plaque psoriasis. Both medications have been shown to be effective in reducing symptoms and improving quality of life for people with psoriasis. Infliximab is a TNF-alpha inhibitor, which works by blocking the action of a protein that contributes to inflammation in the body. It is typically administered intravenously. Risankizumab is an interleukin-23 inhibitor, which targets a different protein involved in inflammation. It is administered subcutaneously. In clinical trials, both infliximab and risankizumab have been shown to be effective at improving skin clearance and reducing symptoms of psoriasis. However, risankizumab has been found to be more effective at maintaining clearance of skin after treatment has been discontinued. In terms of safety, both medications have similar safety profiles, and can cause similar side effects such as infections and allergic reactions. However, as with any medication, individual responses may vary. It is important to consult with a healthcare professional to determine the most appropriate treatment for a specific individual.

13. **Comparative Efficacy** and Relative Ranking of Biologics and Oral Therapies for Moderate-to-Severe Plaque **Psoriasis**: A Network Meta-analysis. Armstrong AW, Soliman AM, Betts KA, Wang Y, Gao Y, Puig L, Augustin M. Dermatol Ther (Heidelb). 2021 Jun;11(3):885-905. doi: 10.1007/s13555-021-00511-1.

Conclusion of the MA: Ixekizumab, risankizumab, and brodalumab had the highest short-term efficacy, and risankizumab had the highest long-term efficacy.
Query: Among the following drugs : ixekizumab, risankizumab, brodalumab, guselkumab, secukinumab, infliximab, certolizumab pegol, ustekinumab, adalimumab, tildrakizumab, etanercept, apremilast, dimethyl fumarate, which ones have the highest short-term efficacy for moderate-to-severe plaque psoriasis?
ChatGPT output: Ixekizumab, risankizumab, brodalumab, guselkumab, secukinumab, and tildrakizumab have the highest short-term efficacy for Moderate-to-Severe Plaque Psoriasis. These medications are known as biologic drugs and they work by targeting specific molecules in the immune system that contribute to the development of psoriasis. They are typically administered as injections or infusions, and can be very effective in reducing inflammation and clearing up skin lesions. However, it's important to note that the short term efficacy can vary depending on the patient, and it's always best to consult with a dermatologist to determine the most appropriate treatment plan for an individual.
Query: Among the following drugs : risankizumab, brodalumab, guselkumab, ixekizumab, secukinumab, ustekinumab, adalimumab, etanercept, which ones have the highest long-term efficacy for moderate-to-severe plaque psoriasis?
ChatGPT output: Ixekizumab, secukinumab, and ustekinumab have been shown to have the highest long-term efficacy for moderate-to-severe plaque psoriasis.

14. Network meta-analysis of biologic treatments for **psoriasis** using absolute **Psoriasis** Area and Severity Index values ≤1, 2, 3 or 5 derived from a statistical conversion method. Mrowietz U, Warren RB, Leonardi CL, Saure D, Petto H, Hartz S, Dossenbach M, Reich K. J Eur Acad Dermatol Venereol. 2021 May;35(5):1161-1175.

Conclusion of the MA: The most effective treatment regimens based on both absolute and relative PASI NMAs were brodalumab 210 mg every 2 weeks and ixekizumab 80 mg every 2 weeks, followed by guselkumab 100 mg every 8 weeks and risankizumab 150 mg every 12 weeks.
Query: Compare the efficacy of infliximab, ustekinumab, certolizumab pegol, ixekizumab, brodalumab, guselkumab, risankizumab, secukinumab, etanercept, tildrakizumab and adalimumab in the treatment of psoriasis.
ChatGPT output: Infliximab, ustekinumab, certolizumab pegol, ixekizumab, brodalumab, guselkumab, risankizumab, secukinumab, etanercept, tildrakizumab, and adalimumab are all biologics that have been FDA approved for the treatment of psoriasis. These drugs are designed to target specific molecules involved in the immune response that contribute to the development of psoriasis. Infliximab, ustekinumab, and secukinumab target IL-12 and IL-23, whereas ixekizumab, brodalumab, guselkumab, risankizumab, tildrakizumab target IL-17A. Certolizumab pegol targets TNF-alpha, and etanercept, adalimumab targets TNF-alpha and also TNF-beta. All these drugs have been shown to be effective in the treatment of psoriasis. However, the specific efficacy of each drug can vary depending on the individual patient and the severity of their condition. Some of these drugs may also be more effective for certain subtypes of psoriasis or in combination with other treatments. It's important to note that while these drugs are effective, they do have different side effect profile and may not be suitable for all patients, so it's best to consult with a medical professional or a dermatologist for personalized advice on the best treatment options for psoriasis.

15. Number needed to treat for interleukin inhibitors approved for the treatment of moderate-to-severe plaque psoriasis in Italy. Ravasio R, Costanzo A, Antonelli S, Maiorino A, Losi S. Glob Reg Health Technol Assess 2021 Apr 15;8:53-57.

Conclusion of the MA: The findings show that ixekizumab is the most effective option (NNT) for the treatment of moderate-to-severe plaque psoriasis.
Query: Among these biologics : brodalumab, ixekizumab, secukinumab, guselkumab, risankizumab, tildrakizumab and ustekinumab, which one is the most effective for the treatment of moderate to severe plaque psoriasis?
ChatGPT output: The most effective biologic for the treatment of moderate to severe plaque psoriasis will depend on individual patient characteristics and response to treatment. Studies have shown that several biologic drugs, including brodalumab, ixekizumab, secukinumab, guselkumab, risankizumab, tildrakizumab and ustekinumab, can effectively treat moderate to severe plaque psoriasis with high levels of efficacy. It is difficult to determine which one is the most effective as the results can vary based on the study population, design, and criteria for evaluating outcomes. Additionally, the best option for a specific patient will depend on individual characteristics and response to treatment. It is recommended to consult a healthcare professional for personalized treatment recommendations and to discuss the potential benefits and risks of each treatment option.
Query: Among these biologics : brodalumab, ixekizumab, secukinumab, guselkumab, risankizumab, tildrakizumab and ustekinumab, which one has the most number needed to treat for the treatment of moderate to severe plaque psoriasis?
ChatGPT output: The "number needed to treat" (NNT) is a statistical measure that indicates how many patients need to be treated with a particular medication to achieve a positive response in one patient. The NNT can vary based on the study population, design, and criteria for evaluating outcomes. Unfortunately, I don't have access to the latest data on NNT for biologic therapies for the treatment of moderate to severe plaque psoriasis. The NNT for each biologic can vary based on the study population, design, and criteria for evaluating outcomes. It is recommended to consult a healthcare professional for personalized treatment recommendations and to discuss the potential benefits and risks of each treatment option.
Query: Rank these biologics : brodalumab, ixekizumab, secukinumab, guselkumab, risankizumab, tildrakizumab and ustekinumab, as the most effective treatment of moderate to severe psoriasis.
ChatGPT output: The most effective biologic treatment for moderate to severe psoriasis is a matter of clinical judgement and can vary from patient to patient. However, some biologics are generally considered more effective than others for treating psoriasis. Here is a general overview of the efficacy of these biologics for moderate to severe psoriasis:
1. Ixekizumab and secukinumab are considered to be highly effective in treating moderate to severe psoriasis.
2. Ustekinumab and guselkumab are also highly effective in treating moderate to severe psoriasis.
3. Tildrakizumab is a moderately effective treatment for moderate to severe psoriasis.
4. Brodalumab is considered to be an effective treatment for moderate to severe psoriasis, although its long-term safety profile is still being evaluated.
5. The efficacy of risankizumab in treating moderate to severe psoriasis is still being studied.

It is important to note that this is a general overview and that the efficacy of these drugs may vary between individuals and conditions. It is recommended to consult with a healthcare professional to determine the best treatment plan for your specific needs.

16. <u>A Systematic Review With Network Meta-Analysis of the Available Biologic Therapies for Psoriatic Disease Domains.</u> Torres T, Barcelos A, Filipe P, Eurico Fonseca J. Front Med (Lausanne) 2021 Jan 15;7:618163.

Conclusion of the MA: Secukinumab and ixekizumab were the treatments with the highest probability of reaching both PASI100 and ACR70 outcomes. Due to the lack of a standard

evaluation of outcomes of the other psoriatic disease domains, a network meta-analysis for all the domains was not possible to perform.
Query: Which biologic among brodalumab, ixekizumab, secukinumab, guselkumab, risankizumab, tildrakizumab, ustekinumab, briakinumab, adalimumab, certolizumab pegol and etanercept has the highest probability of reaching PASI100 and ACR70 outcomes?
ChatGPT output: The probability of reaching PASI100 (a skin clearance score of 100%) and ACR70 (a 70% improvement in American College of Rheumatology criteria) outcomes with different biologic treatments can vary based on the study population, design, and criteria for evaluating outcomes. Studies have shown that several biologic drugs, including brodalumab, ixekizumab, secukinumab, guselkumab, risankizumab, tildrakizumab, ustekinumab, briakinumab, adalimumab, certolizumab pegol and etanercept, can effectively treat moderate to severe plaque psoriasis with high levels of efficacy and reach PASI100 and ACR70 outcomes in a significant proportion of patients. It is recommended to consult a healthcare professional for personalized treatment recommendations and to discuss the potential benefits and risks of each treatment option.
Query: Rank these biologics: brodalumab, ixekizumab, secukinumab, guselkumab, risankizumab, tildrakizumab, ustekinumab, briakinumab, adalimumab, certolizumab pegol and etanercept as the most likely to reach PASI100 and ACR70 outcomes.
ChatGPT output: The ability of biologics to reach PASI100 (100% improvement in the Psoriasis Area and Severity Index) and ACR70 (70% improvement in the American College of Rheumatology criteria) outcomes can vary between individuals and is dependent on a number of factors, including the severity of the condition, the presence of comorbidities, and the patient's response to treatment. However, here is a general overview of the biologics and their likelihood to reach these outcomes:
1. Ixekizumab, secukinumab, guselkumab, and brodalumab are considered to be highly effective in reaching PASI100 and ACR70 outcomes.
2. Ustekinumab and adalimumab are also effective in reaching these outcomes, although to a slightly lesser extent.
3. Certolizumab pegol and etanercept have been shown to be moderately effective in reaching PASI100 and ACR70 outcomes.
4. Briakinumab and risankizumab are still being studied and their ability to reach these outcomes is still being evaluated.
5. Tildrakizumab has been shown to be effective in reaching PASI100, but its ability to reach ACR70 outcomes is still being evaluated.
It is important to note that this is a general overview and the efficacy of these drugs may vary between individuals and conditions. It is recommended to consult with a healthcare professional to determine the best treatment plan for your specific needs.

17. Additional queries

Additional query: Do you have access to paid scientific content?
ChatGPT ouput: No, I do not have direct access to paid scientific content.
Additional query: What is your knowledge cut-off?
ChatGPT ouput: My knowledge cut-off is 2021, which means that the information and data I have been trained on goes up to that year and does not include any events or developments that have occurred since then. It's important to keep this in mind when asking questions, especially if you are seeking information about recent events or advancements.
Additional query: If you have to compare the efficacy and tolerance of different treatments for a specific disease what are your strenghts and weaknesses as compared to meta-analyses?
ChatGPT output: As a language model, my strengths include the ability to quickly and accurately process large amounts of data from a variety of sources, such as medical journals

and clinical studies. I can also provide detailed and up-to-date information on a wide range of treatments for a specific disease. My main weakness is that I am not able to independently assess the quality or validity of the sources I am using, so the information I provide may be biased or inaccurate if the sources themselves are of low quality. Additionally, I am not able to conduct a meta-analysis, which is a statistical method used to combine the results of multiple studies in order to provide a more robust and reliable estimate of treatment efficacy and tolerability. In summary, while I can provide a wide range of information on treatments for a specific disease, my information is only as reliable as the sources I am using and I am not able to conduct a meta-analysis. To gain the most accurate and reliable information, it would be best to consult with a healthcare professional or conduct a meta-analysis by a specialist.

Additional query: When comparing the efficacy and tolerance of different treatments for a specific disease and when considering the relative strenghts and weaknesses of meta-analyses and ChatGPT, are your results more valuable in real-life conditions than those of meta-analyses.

ChatGPT output: Meta-analyses are valuable tools for synthesizing the results of multiple studies on a specific topic, and can provide a more comprehensive understanding of the efficacy and tolerance of different treatments for a specific disease. However, ChatGPT, as a language model, is not able to conduct meta-analyses or provide information about efficacy and tolerance of treatments. It can provide information based on the knowledge it has been trained on, but it cannot provide an analysis of studies or real-life conditions. Therefore, the results obtained from ChatGPT cannot be considered more valuable than those of meta-analyses in terms of evaluating treatment efficacy and tolerance.

Additional query: What algorithms do you use to analyze the searched data? How do you choose which information to use, which data to prioritize, and how do you summarize all these data?

ChatGPT output: As an AI language model developed by OpenAI, I use a transformer-based architecture to process and generate text. The model is trained on a diverse range of internet text, so it can generate responses based on the input it receives. When I receive a question, I analyze it by breaking it down into individual tokens and then generating a representation of each token, called an embedding. The embeddings are then fed through several layers of the network, where they are transformed and combined with other information from the input to generate a probability distribution over the possible answers. To determine which information to use, the model uses its training data to generate a probability distribution over possible responses, and chooses the most likely response based on that distribution. In general, the model tries to balance between being relevant to the input, consistent with what it has learned during its training, and being coherent and natural-sounding. As for summarizing data, the model doesn't have a direct mechanism for summarization. However, it can generate concise and clear answers based on the information it has learned during its training and the input it receives. If the input includes a lot of information, the model will try to extract the most important details and present them in a concise and informative manner.

Additional query: Is your dataset locked right after the queries are sent ?

ChatGPT output: No, the dataset that I was trained on is not locked after the queries are sent. As a machine learning model, I don't have any control over the data I was trained on, or its availability. The training data is stored and maintained by OpenAI, and they have the ability to update it and make changes as needed. However, it's important to note that I am not able to access new information or updates to the training data in real-time. The knowledge that I have is based on the training data that was available at the time of my training, and my responses are generated based on that knowledge.

Additional query: How are you coded by your developers?

ChatGPT output: I was developed by OpenAI using a type of deep learning architecture called a Transformer. The Transformer architecture is based on attention mechanisms, which allow

the model to dynamically focus on different parts of the input when generating its outputs. My training data consists of a large corpus of text from the internet, and the model was trained using a variant of the transformer architecture called GPT-3 (Generative Pretrained Transformer-3). During training, the model learned to predict the next word in a sequence given the previous words, and this training process allowed it to learn patterns in language and build up a knowledge base of facts and concepts. The code for my model was implemented using a combination of PyTorch and Python. The model was trained on a cluster of powerful GPUs, which allowed for fast training times and allowed the model to learn from a large amount of data. Once the model was trained, it was fine-tuned on specific tasks, such as answering questions or generating text, to make it better at those tasks. The fine-tuning process involved adjusting the model's parameters to make it better at generating outputs that are relevant and accurate for a specific task.

Additional query: why do your answers change depending on the day we send you queries?

ChatGPT output: As an AI language model, I was trained on a large corpus of text from the internet, but my training data has a cutoff date of 2021, so I don't have information on events or developments that have occurred after that time. Additionally, the training data that I was trained on may change over time as OpenAI updates and improves it. While I can generate answers based on the information that I was trained on, my responses can still change depending on the context of the question and other factors, such as the specific fine-tuning that I was subjected to. It's also possible that the way that I generate answers has been updated by OpenAI since my training, which could result in changes to the answers I provide. For these reasons, it's possible that my answers may change over time and vary between different instances of my use.
